# Supplementary material for: Predicting trends in the quality of state-of-the-art neural networks without access to training or testing data
Source: Nat Commun. 2021 Jul 5;12:4122. doi: 10.1038/s41467-021-24025-8 (PMC8257709; doi:10.1038/s41467-021-24025-8)
Supplement: Supplementary file 1 — Supplementary Information [file 41467_2021_24025_MOESM1_ESM.pdf]

Supplementary Information: "Predicting trends in the quality of state-of-the-art neural networks without access to training or testing data" by Martin, Peng, and Mahoney

Supplementary Note 1: Supplementary Details

Reproducing the CV and NLP Sections. We provide a github repository for this paper that includes Jupyter notebooks that fully reproduce all results (as well as many other results) [7]. All results have been produced using the `WeightWatcher` tool) [6]. The ImageNet and OpenAI GPT pretrained models are provided in the current pyTorch [20] and Huggingface [29] distributions.

Reproducing Figure 5, for the Distiller Model. In the `distiller` folder of our github repo, we provide the original Jupyter Notebooks, which use the Intel `distiller` framework [27]. Figure 5 is from the `''...-Distiller-ResNet20.ipynb''` notebook (see Supplementary Table 1). For completeness, we provide both the results described here, as well as additional results on other pretrained and distilled models using the `WeightWatcher` tool.

Reproducing Table 3. The reader may regenerate all of the results of the CV Section. `WeightWatcher` results using the Google Colab Jupyter notebooks (in the `ww-colab` folder) and the `WeightWatcher` tool, and/or simply reproduce Table 3, as well as Supplementary Tables 3-5 and Supplementary Figures 1-9, using the Jupyter notebooks (shown in Supplementary Table 1), and the pre-computed `WeightWatcher` datasets (in the `data/osmr` folder). The pretrained models, trained on ImageNet-1K and the other datasets, are taken from the pyTorch models in the `omsr/imgclsmob` "Sandbox for training convolutional networks for computer vision" github repository [5]. The full `WeightWatcher` results are provided in the datasets: `[data/osmr/data...xlsx]`, last generated in January 2020, using the Google Colab notebooks: `[ww_colab/ww_colab...ipynb]`, and `WeightWatcher` version ww0.2.7. Results can be recomputed using the current version (ww0.4.1) using the `ww2x=True` back compatability option, although note the pretrained models must be downloaded and may have changed slightly. The data files currently provided are analyzed with the `OSMR-Analysis.ipynb` python Juyter Notebook, which runs all regressions and which tabulates the results presented in Table 3 and generates the figures in Supplementary Figures 1-9 and Supplementary Tables 3-5.

We attempt to run linear regressions for all pyTorch models for each architecture series for all datasets provided. There are over 450 models in all to consider, and we note that the `osmr/imgclsmob` repository is constantly being updated with new models. We omit the results for CUB-200-2011, Pascal-VOC2012, ADE20K, and COCO datasets, as there are fewer than 15 models for those datasets. Also, we filter out regressions with fewer than 5 datapoints.

We remove the following outliers, as identified by visual inspection: `efficient_b0,_b2`. We also remove the entire `cifar100 ResNeXT` series, which is the only example to show no trends with the norm metrics. The final architecture series used are shown in Supplementary Table 2, with the number of models in each.

Tables and figures summarizing this analysis (in a more fine-grained way than provided by Table 3) are presented next.

Supplementary Note 2: Supplementary Tables and Figures

Here, we present a more detailed discussion of our large-scale analysis of hundreds of models, which were summarized in Table 3. We ran the `WeightWatcher` tool (version 0.2.7) [6] on numerous pretrained models taken from the `OSMR/imgclsmob Sandbox` github repository of pretrained CV

Table	Figure	Jupyter Notebook
1	2	WeightWatcher-VGG.ipynb
1	3(a)	WeightWatcher-ResNet.ipynb
1	3(b)	WeightWatcher-ResNet-1K.ipynb
1	4(a)	WeightWatcher-VGG.ipynb
1	4(b)	WeightWatcher-ResNet.ipynb
1	4(c)	WeightWatcher-DenseNet.ipynb
	5	WeightWatcher-Intel-Distiller-ResNet20.ipynb
2	6	WeightWatcher-OpenAI-GPT.ipynb
2	7, 8	WeightWatcher-OpenAI-GPT2.ipynb
3,7,8,9	Appendix	OSMR-Analysis.ipynb

Supplementary Table 1: Jupyter notebooks used to reproduce all results in the CV and NLP Sections.

DNN models [5], performing OLS (Ordinary Least Squares) regressions for every dataset and architecture series listed in Supplementary Table 2. Table 3 summarized the overall results, and Supplementary Figures 1–9 below present a more detailed visual summary, which enables model diagnostics.

For each Figure, each row of subfigures considers a given pretrained model and dataset, depicting the average Norm-based and Power Law metrics—Log Frobenius norm ($\langle \log \|\mathbf{W}\|_F^2 \rangle$), Log Spectral norm ($\langle \log \|\mathbf{W}\|_\infty^2 \rangle$), Weighted Alpha ($\hat{\alpha}$), and Log α-Norm ($\langle \log \|\mathbf{X}\|_\alpha^\alpha \rangle$)—against the Top1 Test Accuracy, as reported in the github repository README file [5], along with a shaded area representing the 95% confidence bound. For each regression, we report the RMSE, the R2 regresssion metric, and the Kendal-τ rank correlation metric in the title of each subfigure. We also present these same numerical values in Supplementary Table 3, Supplementary Table 4, and Supplementary Table 5, respectively. To reproduce these Figures and Tables, see the `OSMR-Analysis.ipynb` python Jupyter Notebook, as listed in Supplementary Table 1. (These are provided in the github repo accompanying this paper.) The reader may regenerate these Figures and Tables, as well as more fine grained results, by rerunning the `OSMR-Analysis.ipynb` python Jupyter Notebook (see Supplementary Table 1), which analyzes the precomputed data in the `df_all.xlsx` file. This repository also contains the original `Google Colab` notebooks, run in January 2020, which download the pretrained models and run the `WeightWatcher` tool on them. The reader may also run the `WeightWatcher` locally on each of the pretrained models, such as the ResNet models, trained on the ImageNet-1K dataset, using the `WeightWatcher-ResNet-1K.ipynb` notebook. (We should note, however, that the publicly-available versions of these models may have changed slightly, giving slightly different results). Our final analysis includes 108 regressions in all. See Supplementary Figure 1–9 for more details.

From these Figures, we recognize fits of varying quality, ranging from remarkably good to completely uncorrelated. Starting with some of the best, consider the Imagenet-1K PreResNet results. For example, Supplementary Figure 1(j) shows the Log Spectral norm, which has a rather large $RMSE = 3.93$, and rather small $R2 = 0.36$, and Kendal-$\tau = 0.54$, and which has 6 out of 13 points outside the 95% confidence bands. In contrast, the Log Frobenius norm in Supplementary Figure 1(l) has a much smaller $RMSE = 1.93$, a much larger $R2 = 0.85$, and Kendal-$\tau = 0.87$, and has only 2 points outside the 95% confidence bands. For examples of lower quality fits, consider the SqueezeNext results, as shown Supplementary Figures 3(j) and 3(l). The Log Spectral norm appears visually anti-correlated with the test accuracies (as it is with ShuffleNet, in Supplementary Figure 2(b)). It has a very large 95% confidence band, with only 2 points close to the regression line, a large RMSE, $R2 = 0.07$ (i.e., near zero), and small Kendal-

Architecture	# of Models	Datasets				
	total	imagenet-1k	cifar-10	cifar-100	svhn	cub-200-2011
EfficientNet	20	20	0	0	0	0
ResNet	48	19	8	8	7	6
PreResNet	14	14	0	0	0	0
VGG/BN-VGG	12	12	0	0	0	0
ShuffleNet	12	12	0	0	0	0
DLA	10	10	0	0	0	0
HRNet	9	9	0	0	0	0
DRN-C/DRN-D	7	7	0	0	0	0
SqueezeNext/SqNxt	6	6	0	0	0	0
ESPNetv2	5	5	0	0	0	0
SqueezeNet/SqueezeResNet	4	4	0	0	0	0
IGCV3	4	4	0	0	0	0
ProxylessNAS	4	4	0	0	0	0
DIA-ResNet/DIA-PreResNet	24	0	8	8	8	0
SENet/SE-ResNet	20	0	5	5	4	6
WRN	8	0	0	4	4	0
ResNeXt	4	0	0	0	4	0
total per dataset	211	126	21	25	27	12

Supplementary Table 2: Number of models for each architecture–dataset pair used in our large-scale analysis.

$\tau = 0.33$. The Log Frobenius norm is (as always) positively-correlated with test accuracies, but with $R2 = 0.43$, it show some linear correlation, and a reasonable Kendal-$\tau = 0.73$, showing moderately strong rank correlation.

Many more such conclusions can be drawn by examining these Tables and Figures and reproducing the results from our publicly-available repo.

Supplementary Note 3: Additional Details on HT-SR Theory

The original work on HT-SR Theory [1, 2, 3] considered DNNs including AlexNet and InceptionV3 (as well as DenseNet, ResNet, and VGG), and it showed that for nearly every $\mathbf{W}$, the (bulk and tail) of the ESDs can be fit to a truncated PL and the PL exponents α nearly all lie within the range $\alpha \in (1.5, 5)$. Our meta-analysis, the main results of which are summarized in this paper, has shown that these results are ubiquitous. For example, upon examining nearly 10,000 layer weight matrices $\mathbf{W}_{l,i}$ across hundreds of different modern pre-trained DNN architectures, the ESD of nearly every $\mathbf{W}$ layer matrix can be fit to a truncated PL: $70 - 80\%$ of the time, the fitted PL exponent α lies in the range $\alpha \in (2, 4)$; and $10 - 20\%$ of the time, the fitted PL exponent α lies in the range $\alpha < 2$. Of course, there are exceptions: in any real DNN, the fitted α may range anywhere from ~ 1.5 to 10 or higher (and, of course, larger values of α may indicate that the PL is not a good model for the data). Still, overall, in nearly all large, pre-trained DNNs, the correlations in the weight matrices exhibit a remarkable Universality, being both Heavy Tailed, and having small—but not too small—PL exponents.

Dataset	Model	$\langle \log \|\cdot\|_F^2 \rangle$	$\langle \log \|\cdot\|_\infty^2 \rangle$	$\hat{\alpha}$	$\langle \log \|\cdot\|_\alpha^\alpha \rangle$
imagenet-1k	EfficientNet	1.64	**1.11**	1.60	1.58
imagenet-1k	ResNet	2.52	3.29	**1.88**	2.00
imagenet-1k	PreResNet	2.57	3.93	**1.90**	1.93
imagenet-1k	VGG	1.11	**0.91**	1.57	1.48
imagenet-1k	ShuffleNet	5.95	9.46	4.42	**4.30**
imagenet-1k	DLA	4.79	**3.02**	3.94	4.06
imagenet-1k	HRNet	0.64	0.77	**0.36**	**0.36**
imagenet-1k	DRN-C	0.77	0.81	**0.64**	0.69
imagenet-1k	SqueezeNext	4.68	4.62	3.65	**3.64**
imagenet-1k	ESPNetv2	3.71	3.84	**1.37**	1.59
imagenet-1k	SqueezeNet	0.33	0.33	**0.26**	0.29
imagenet-1k	IGCV3	1.39	9.37	2.91	**1.04**
imagenet-1k	ProxylessNAS	**0.44**	0.51	0.53	0.51
cifar-10	ResNet	0.56	0.55	**0.53**	**0.53**
cifar-10	DIA-ResNet	**0.22**	0.28	0.53	0.56
cifar-10	SENet	0.30	0.30	**0.20**	**0.20**
cifar-100	ResNet	2.03	2.12	**1.75**	**1.75**
cifar-100	DIA-ResNet	**0.60**	1.17	0.96	1.01
cifar-100	SENet	0.60	0.65	**0.51**	**0.51**
cifar-100	WRN	0.37	0.44	0.26	**0.25**
svhn	ResNet	0.20	0.20	**0.15**	0.16
svhn	DIA-ResNet	0.07	**0.06**	0.13	0.13
svhn	SENet	**0.04**	0.07	0.05	0.05
svhn	WRN	**0.07**	0.08	**0.07**	**0.07**
svhn	ResNeXt	**0.06**	**0.06**	0.11	0.09
cub-200-2011	ResNet	0.45	**0.42**	1.79	1.79
cub-200-2011	SENet	**1.03**	1.13	1.36	1.40

Supplementary Table 3: RMSE results for our analysis of all CV models in Table 3.

Dataset	Model	$\langle \log \|\cdot\|_F^2 \rangle$	$\langle \log \|\cdot\|_\infty^2 \rangle$	$\hat{\alpha}$	$\langle \log \|\cdot\|_\alpha^\alpha \rangle$
imagenet-1k	EfficientNet	0.65	**0.84**	0.67	0.67
imagenet-1k	ResNet	0.77	0.61	**0.87**	0.86
imagenet-1k	PreResNet	0.73	0.36	**0.85**	**0.85**
imagenet-1k	VGG	0.63	**0.75**	0.27	0.35
imagenet-1k	ShuffleNet	0.63	0.06	0.80	**0.81**
imagenet-1k	DLA	0.11	**0.65**	0.40	0.36
imagenet-1k	HRNet	0.89	0.85	**0.97**	**0.97**
imagenet-1k	DRN-C	0.81	0.79	**0.87**	0.85
imagenet-1k	SqueezeNext	0.05	0.07	0.42	**0.43**
imagenet-1k	ESPNetv2	0.42	0.38	**0.92**	0.89
imagenet-1k	SqueezeNet	0.01	0.00	**0.38**	0.26
imagenet-1k	IGCV3	0.98	0.12	0.92	**0.99**
imagenet-1k	ProxylessNAS	**0.68**	0.56	0.53	0.58
cifar-10	ResNet	0.58	0.59	**0.62**	0.61
cifar-10	DIA-ResNet	**0.96**	0.93	0.74	0.71
cifar-10	SENet	0.91	0.91	**0.96**	**0.96**
cifar-100	ResNet	0.61	0.58	**0.71**	**0.71**
cifar-100	DIA-ResNet	**0.96**	0.85	0.90	0.89
cifar-100	SENet	0.97	0.96	**0.98**	**0.98**
cifar-100	WRN	0.32	0.04	0.66	**0.69**
svhn	ResNet	0.69	0.70	**0.82**	0.81
svhn	DIA-ResNet	0.94	**0.95**	0.78	0.77
svhn	SENet	**0.99**	0.96	0.98	0.98
svhn	WRN	0.13	0.10	0.20	**0.21**
svhn	ResNeXt	0.87	**0.90**	0.64	0.75
cub-200-2011	ResNet	0.94	**0.95**	0.08	0.08
cub-200-2011	SENet	**0.66**	0.59	0.41	0.38

Supplementary Table 4: R2 results for our analysis of all CV models in Table 3.

Dataset	Model	$\langle \log \|\cdot\|_F^2 \rangle$	$\langle \log \|\cdot\|_\infty^2 \rangle$	$\hat{\alpha}$	$\langle \log \|\cdot\|_\alpha^\alpha \rangle$
imagenet-1k	EfficientNet	0.67	**0.79**	0.66	0.66
imagenet-1k	ResNet	0.78	0.70	**0.91**	0.89
imagenet-1k	PreResNet	0.65	0.54	**0.87**	**0.87**
imagenet-1k	VGG	0.73	**0.79**	0.42	0.52
imagenet-1k	ShuffleNet	0.39	0.09	**0.85**	0.82
imagenet-1k	DLA	0.51	**0.82**	0.78	0.69
imagenet-1k	HRNet	0.56	0.44	**0.61**	**0.61**
imagenet-1k	DRN-C	**0.90**	0.81	**0.90**	**0.90**
imagenet-1k	SqueezeNext	0.47	-0.33	**0.73**	**0.73**
imagenet-1k	ESPNetv2	0.00	0.80	**1.00**	**1.00**
imagenet-1k	SqueezeNet	0.33	0.00	**0.67**	**0.67**
imagenet-1k	IGCV3	**1.00**	0.00	**1.00**	**1.00**
imagenet-1k	ProxylessNAS	**0.33**	**0.33**	**0.33**	**0.33**
cifar-10	ResNet	0.64	0.64	**0.71**	**0.71**
cifar-10	DIA-ResNet	0.79	0.50	**0.86**	0.79
cifar-10	SENet	0.74	**0.95**	**0.95**	**0.95**
cifar-100	ResNet	**0.64**	0.57	**0.64**	**0.64**
cifar-100	DIA-ResNet	**0.93**	0.43	**0.93**	**0.93**
cifar-100	SENet	**1.00**	**1.00**	**1.00**	**1.00**
cifar-100	WRN	**0.67**	-0.67	0.00	0.00
svhn	ResNet	**0.81**	0.71	**0.81**	**0.81**
svhn	DIA-ResNet	**0.86**	**0.86**	0.57	0.57
svhn	SENet	**1.00**	**1.00**	0.67	0.67
svhn	WRN	-0.33	-0.33	**0.67**	**0.67**
svhn	ResNeXt	**0.67**	**0.67**	0.33	0.33
cub-200-2011	ResNet	**1.00**	**1.00**	-0.33	-0.33
cub-200-2011	SENet	**0.87**	**0.87**	-0.20	-0.20

Supplementary Table 5: Kendal-tau results for our analysis of all CV models in Table 3.

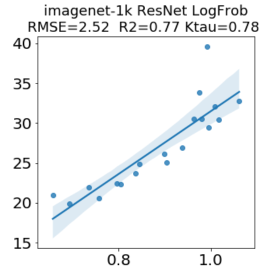

(a) imagenet-1k–ResNet, $\langle \log \|\cdot\|_F \rangle$

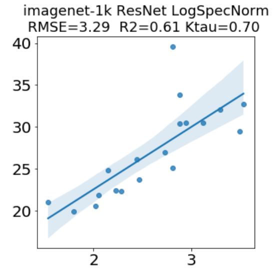

(b) imagenet-1k–ResNet, $\langle \log \|\cdot\|_\infty \rangle$

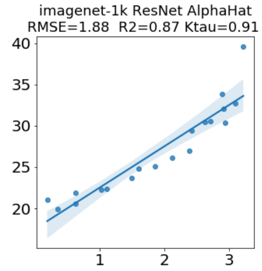

(c) imagenet-1k–ResNet, $\hat{\alpha}$

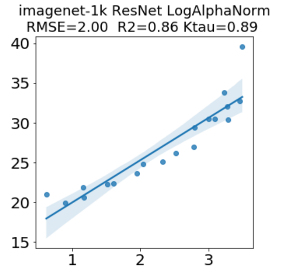

(d) imagenet-1k–ResNet, $\langle \log \|\cdot\|_\alpha^\alpha \rangle$

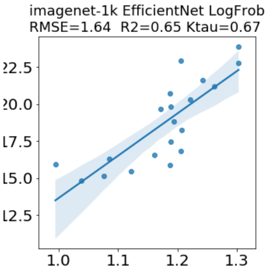

(e) imagenet-1k–EfficientNet, $\langle \log \|\cdot\|_F \rangle$

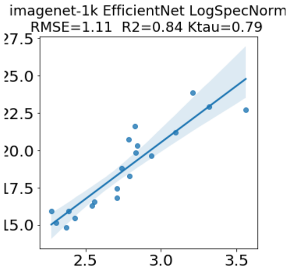

(f) imagenet-1k–EfficientNet, $\langle \log \|\cdot\|_\infty \rangle$

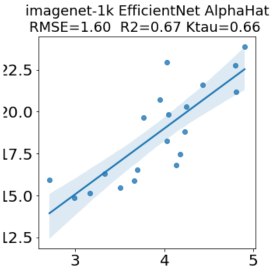

(g) imagenet-1k–EfficientNet, $\hat{\alpha}$

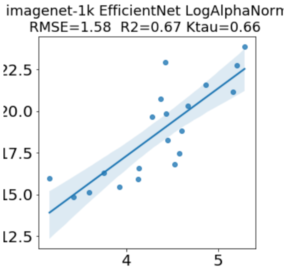

(h) imagenet-1k–EfficientNet, $\langle \log \|\cdot\|_\alpha^\alpha \rangle$

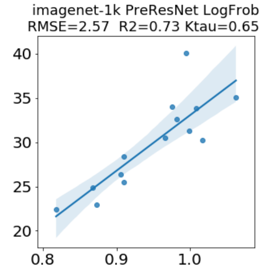

(i) imagenet-1k–PreResNet, $\langle \log \|\cdot\|_F \rangle$

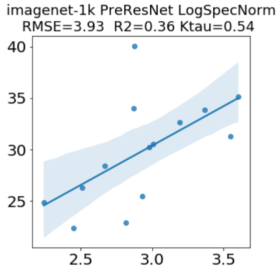

(j) imagenet-1k–PreResNet, $\langle \log \|\cdot\|_\infty \rangle$

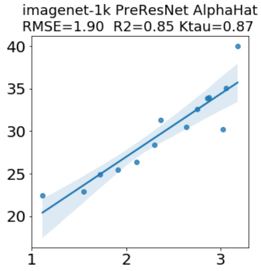

(k) imagenet-1k–PreResNet, $\hat{\alpha}$

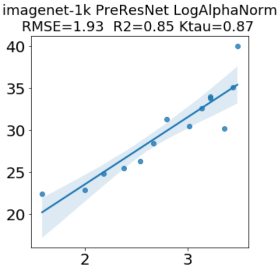

(l) imagenet-1k–PreResNet, $\langle \log \|\cdot\|_\alpha^\alpha \rangle$

Supplementary Figure 1: Regression plots for model-dataset pairs, based on data from Supplementary Table 3, Supplementary Table 4, and Supplementary Table 5. Each row corresponds to a different dataset-model pair: imagenet-1k–ResNet; imagenet-1k–EfficientNet; and imagenet-1k–PreResNet; repsectively. Each column corresponds to a different metric: $\langle \log \|\cdot\|_F \rangle$; $\langle \log \|\cdot\|_\infty \rangle$; $\hat{\alpha}$; and $\langle \log \|\cdot\|_\alpha^\alpha \rangle$; repsectively.

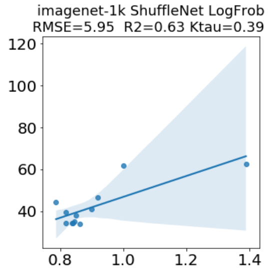

(a) imagenet-1k–ShuffleNet, $\langle \log \| \cdot \|_F \rangle$

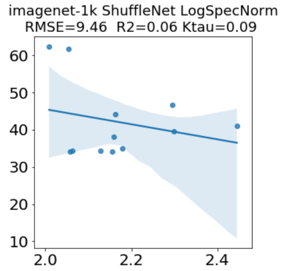

(b) imagenet-1k–ShuffleNet, $\langle \log \| \cdot \|_\infty \rangle$

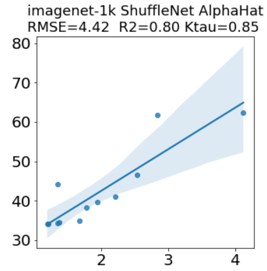

(c) imagenet-1k–ShuffleNet, $\hat{\alpha}$

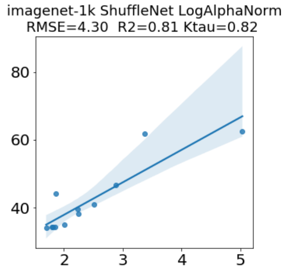

(d) imagenet-1k–ShuffleNet, $\langle \log \| \cdot \|_\alpha^\alpha \rangle$

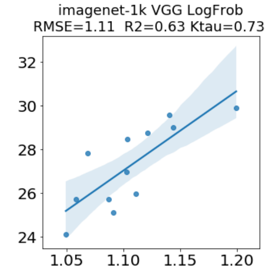

(e) imagenet-1k–VGG, $\langle \log \| \cdot \|_F \rangle$

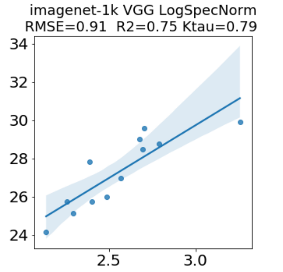

(f) imagenet-1k–VGG, $\langle \log \| \cdot \|_\infty \rangle$

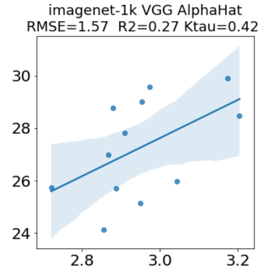

(g) imagenet-1k–VGG, $\hat{\alpha}$

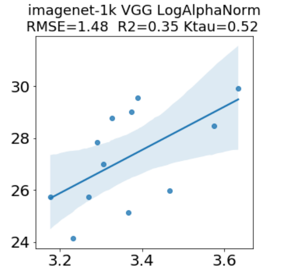

(h) imagenet-1k–VGG, $\langle \log \| \cdot \|_\alpha^\alpha \rangle$

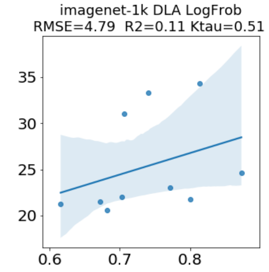

(i) imagenet-1k–DLA, $\langle \log \| \cdot \|_F \rangle$

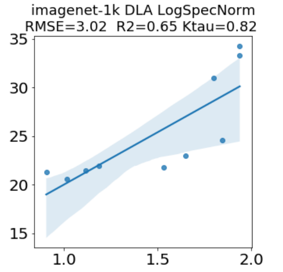

(j) imagenet-1k–DLA, $\langle \log \| \cdot \|_\infty \rangle$

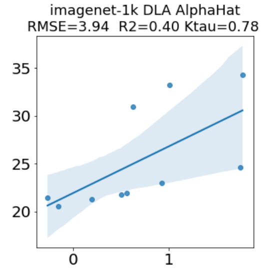

(k) imagenet-1k–DLA, $\hat{\alpha}$

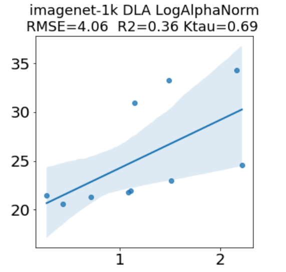

(l) imagenet-1k–DLA, $\langle \log \| \cdot \|_\alpha^\alpha \rangle$

Supplementary Figure 2: Regression plots for model-dataset pairs, based on data from Supplementary Table 3, Supplementary Table 4, and Supplementary Table 5. Each row corresponds to a different dataset-model pair: imagenet-1k–ShuffleNet; imagenet-1k–VGG; and imagenet-1k–DLA; repsectively. Each column corresponds to a different metric: $\langle \log \| \cdot \|_F \rangle$; $\langle \log \| \cdot \|_\infty \rangle$; $\hat{\alpha}$; and $\langle \log \| \cdot \|_\alpha^\alpha \rangle$; repsectively.

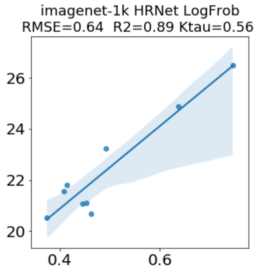

(a) imagenet-1k–HRNet, $\langle \log \|\cdot\|_F \rangle$

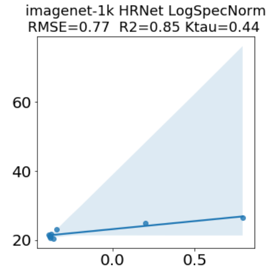

(b) imagenet-1k–HRNet, $\langle \log \|\cdot\|_\infty \rangle$

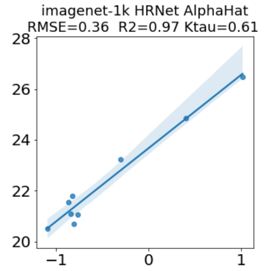

(c) imagenet-1k–HRNet, $\hat{\alpha}$

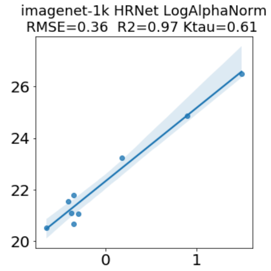

(d) imagenet-1k–HRNet, $\langle \log \|\cdot\|_\alpha^\alpha \rangle$

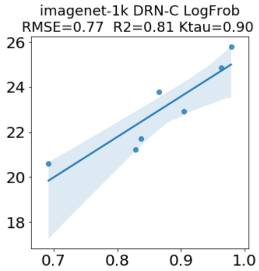

(e) imagenet-1k–DRN-C, $\langle \log \|\cdot\|_F \rangle$

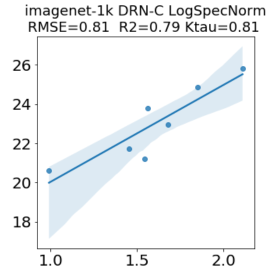

(f) imagenet-1k–DRN-C, $\langle \log \|\cdot\|_\infty \rangle$

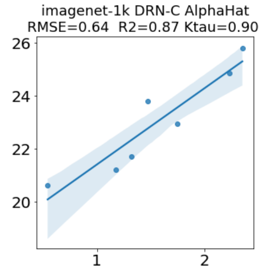

(g) imagenet-1k–DRN-C, $\hat{\alpha}$

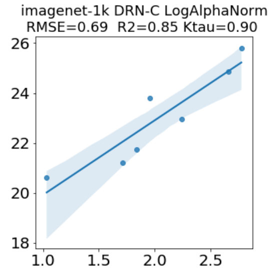

(h) imagenet-1k–DRN-C, $\langle \log \|\cdot\|_\alpha^\alpha \rangle$

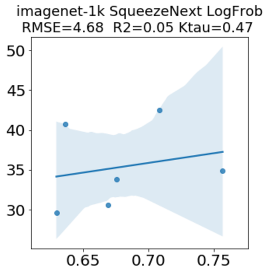

(i) imagenet-1k–SqueezeNext, $\langle \log \|\cdot\|_F \rangle$

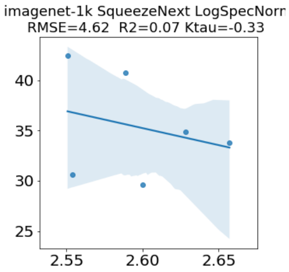

(j) imagenet-1k–SqueezeNext, $\langle \log \|\cdot\|_\infty \rangle$

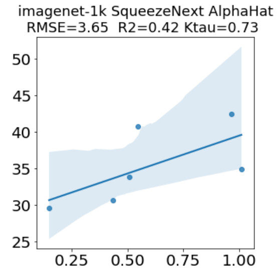

(k) imagenet-1k–SqueezeNext, $\hat{\alpha}$

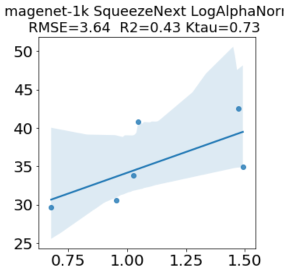

(l) imagenet-1k–SqueezeNext, $\langle \log \|\cdot\|_\alpha^\alpha \rangle$

Supplementary Figure 3: Regression plots for model-dataset pairs, based on data from Supplementary Table 3, Supplementary Table 4, and Supplementary Table 5. Each row corresponds to a different dataset-model pair: imagenet-1k–HRNet; imagenet-1k–DRN-C; and imagenet-1k–SqueezeNext; repsectively. Each column corresponds to a different metric: $\langle \log \|\cdot\|_F \rangle$; $\langle \log \|\cdot\|_\infty \rangle$; $\hat{\alpha}$; and $\langle \log \|\cdot\|_\alpha^\alpha \rangle$; repsectively.

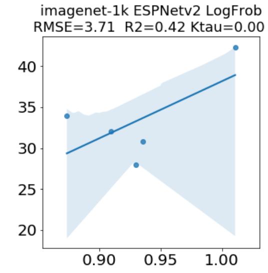

(a) imagenet-1k–ESPNetv2, $\langle \log \| \cdot \|_F \rangle$

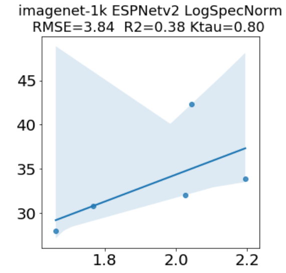

(b) imagenet-1k–ESPNetv2, $\langle \log \| \cdot \|_\infty \rangle$

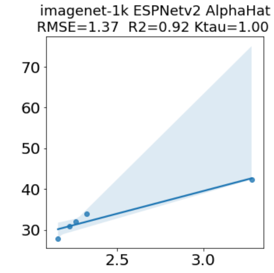

(c) imagenet-1k–ESPNetv2, $\hat{\alpha}$

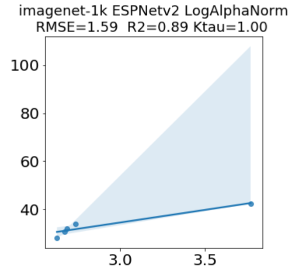

(d) imagenet-1k–ESPNetv2, $\langle \log \| \cdot \|_\alpha^\alpha \rangle$

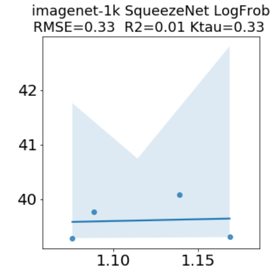

(e) imagenet-1k–SqueezeNet, $\langle \log \| \cdot \|_F \rangle$

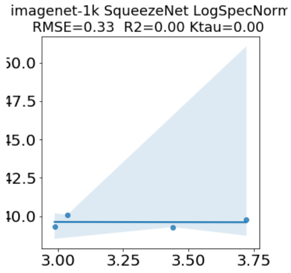

(f) imagenet-1k–SqueezeNet, $\langle \log \| \cdot \|_\infty \rangle$

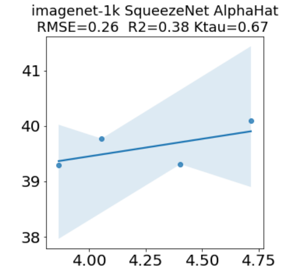

(g) imagenet-1k–SqueezeNet, $\hat{\alpha}$

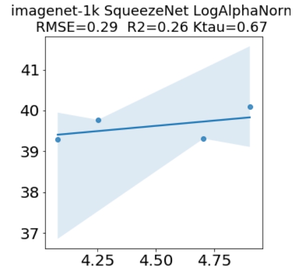

(h) imagenet-1k–SqueezeNet, $\langle \log \| \cdot \|_\alpha^\alpha \rangle$

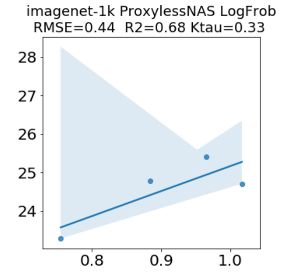

(i) imagenet-1k–ProxylessNAS, $\langle \log \| \cdot \|_F \rangle$

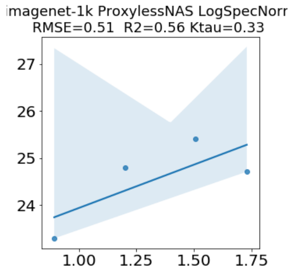

(j) imagenet-1k–ProxylessNAS, $\langle \log \| \cdot \|_\infty \rangle$

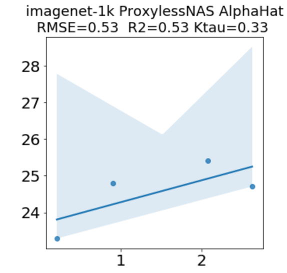

(k) imagenet-1k–ProxylessNAS, $\hat{\alpha}$

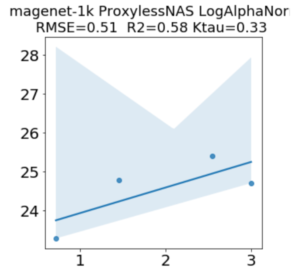

(l) imagenet-1k–ProxylessNAS, $\langle \log \| \cdot \|_\alpha^\alpha \rangle$

Supplementary Figure 4: Regression plots for model-dataset pairs, based on data from Supplementary Table 3, Supplementary Table 4, and Supplementary Table 5. Each row corresponds to a different dataset-model pair: imagenet-1k–ESPNetv2; imagenet-1k–SqueezeNet; and imagenet-1k–ProxylessNAS; repsectively. Each column corresponds to a different metric: $\langle \log \| \cdot \|_F \rangle$; $\langle \log \| \cdot \|_\infty \rangle$; $\hat{\alpha}$; and $\langle \log \| \cdot \|_\alpha^\alpha \rangle$; repsectively.

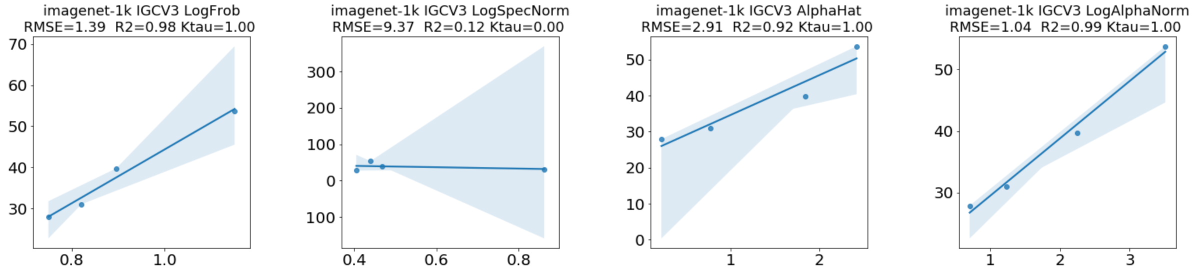

(a) imagenet-1k–IGCV3, $\langle \log \|\cdot\|_F \rangle$ (b) imagenet-1k–IGCV3, $\langle \log \|\cdot\|_\infty \rangle$ (c) imagenet-1k–IGCV3, $\hat{\alpha}$ (d) imagenet-1k–IGCV3, $\langle \log \|\cdot\|_\alpha^\alpha \rangle$

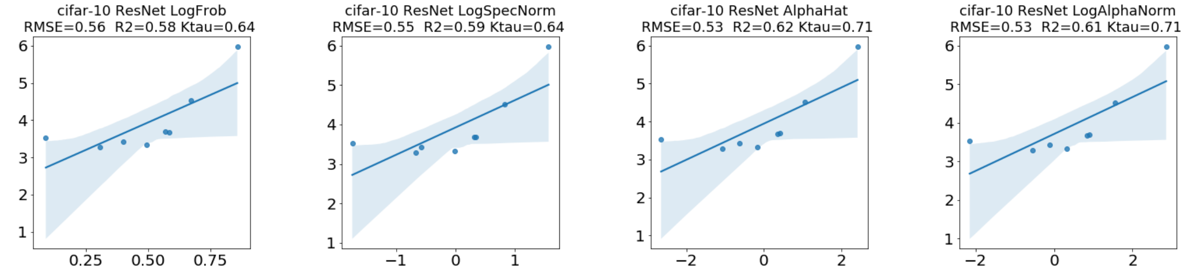

(e) cifar-10–ResNet, $\langle \log \|\cdot\|_F \rangle$ (f) cifar-10–ResNet, $\langle \log \|\cdot\|_\infty \rangle$ (g) cifar-10–ResNet, $\hat{\alpha}$ (h) cifar-10–ResNet, $\langle \log \|\cdot\|_\alpha^\alpha \rangle$

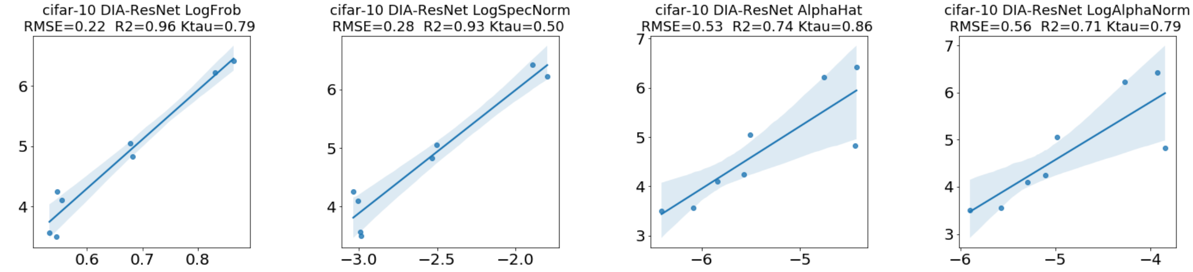

(i) cifar-10–DIA-ResNet, $\langle \log \|\cdot\|_F \rangle$ (j) cifar-10–DIA-ResNet, $\langle \log \|\cdot\|_\infty \rangle$ (k) cifar-10–DIA-ResNet, $\hat{\alpha}$ (l) cifar-10–DIA-ResNet, $\langle \log \|\cdot\|_\alpha^\alpha \rangle$

Supplementary Figure 5: Regression plots for model-dataset pairs, based on data from Supplementary Table 3, Supplementary Table 4, and Supplementary Table 5. Each row corresponds to a different dataset-model pair: imagenet-1k–IGCV3; cifar-10–ResNet; and cifar-10–DIA-ResNet; repsectively. Each column corresponds to a different metric: $\langle \log \|\cdot\|_F \rangle$; $\langle \log \|\cdot\|_\infty \rangle$; $\hat{\alpha}$; and $\langle \log \|\cdot\|_\alpha^\alpha \rangle$; repsectively.

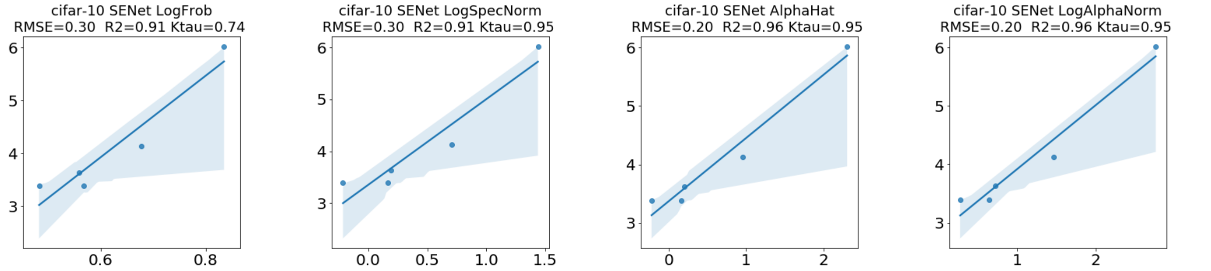

(a) cifar-10–SENet, $\langle \log \|\cdot\|_F \rangle$ (b) cifar-10–SENet, $\langle \log \|\cdot\|_\infty \rangle$ (c) cifar-10–SENet, $\hat{\alpha}$ (d) cifar-10–SENet, $\langle \log \|\cdot\|_\alpha^\alpha \rangle$

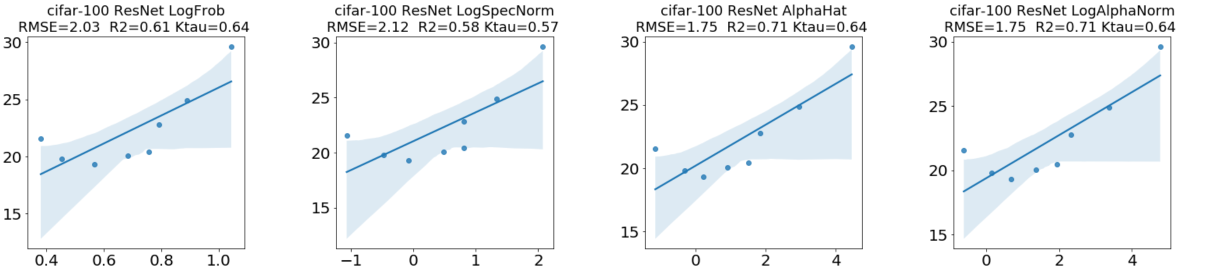

(e) cifar-100–ResNet, $\langle \log \|\cdot\|_F \rangle$ (f) cifar-100–ResNet, $\langle \log \|\cdot\|_\infty \rangle$ (g) cifar-100–ResNet, $\hat{\alpha}$ (h) cifar-100–ResNet, $\langle \log \|\cdot\|_\alpha^\alpha \rangle$

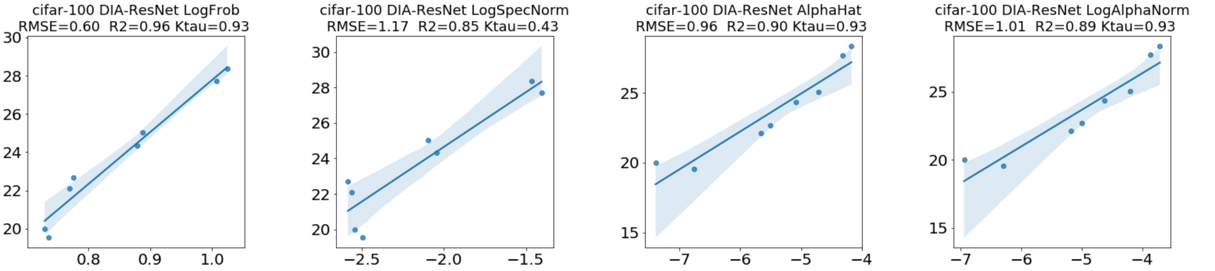

(i) cifar-100–DIA-ResNet, $\langle \log \|\cdot\|_F \rangle$ (j) cifar-100–DIA-ResNet, $\langle \log \|\cdot\|_\infty \rangle$ (k) cifar-100–DIA-ResNet, $\hat{\alpha}$ (l) cifar-100–DIA-ResNet, $\langle \log \|\cdot\|_\alpha^\alpha \rangle$

Supplementary Figure 6: Regression plots for model-dataset pairs, based on data from Supplementary Table 3, Supplementary Table 4, and Supplementary Table 5. Each row corresponds to a different dataset-model pair: cifar-10–SENet; cifar-100–ResNet; and cifar-100–DIA-ResNet; repsectively. Each column corresponds to a different metric: $\langle \log \|\cdot\|_F \rangle$; $\langle \log \|\cdot\|_\infty \rangle$; $\hat{\alpha}$; and $\langle \log \|\cdot\|_\alpha^\alpha \rangle$; repsectively.

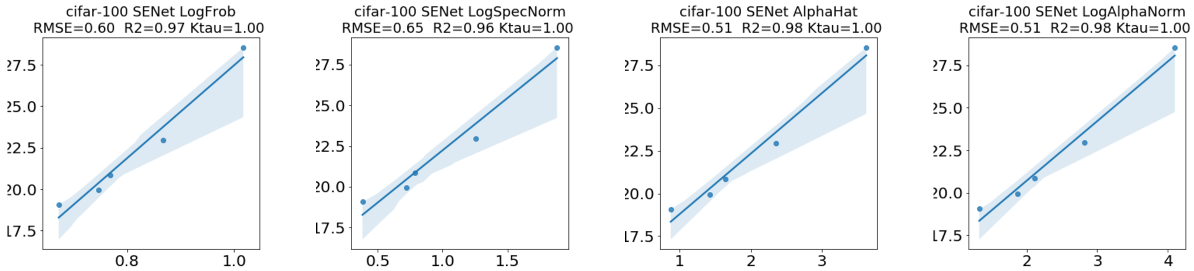

(a) cifar-100–SENet, $\langle \log \|\cdot\|_F \rangle$ (b) cifar-100–SENet, $\langle \log \|\cdot\|_\infty \rangle$ (c) cifar-100–SENet, $\hat{\alpha}$ (d) cifar-100–SENet, $\langle \log \|\cdot\|_\alpha^\alpha \rangle$

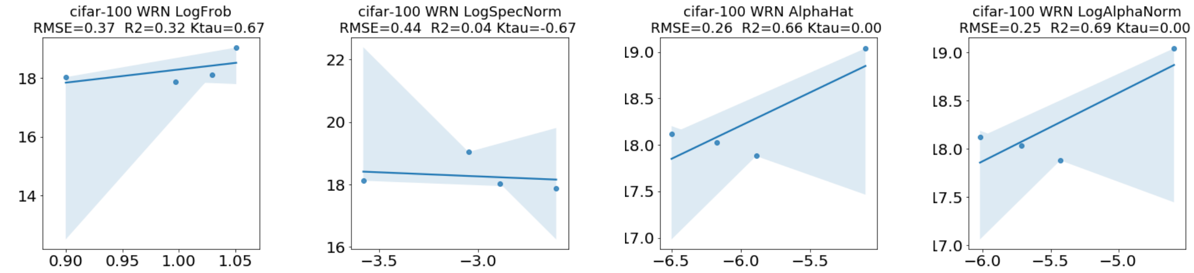

(e) cifar-100–WRN, $\langle \log \|\cdot\|_F \rangle$ (f) cifar-100–WRN, $\langle \log \|\cdot\|_\infty \rangle$ (g) cifar-100–WRN, $\hat{\alpha}$ (h) cifar-100–WRN, $\langle \log \|\cdot\|_\alpha^\alpha \rangle$

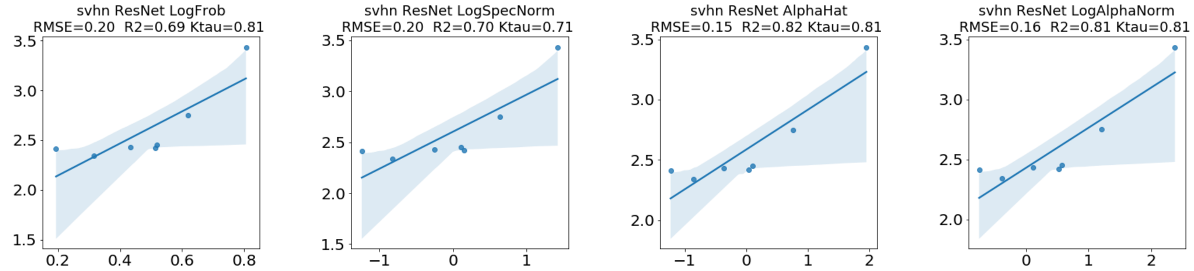

(i) svhn–ResNet, $\langle \log \|\cdot\|_F \rangle$ (j) svhn–ResNet, $\langle \log \|\cdot\|_\infty \rangle$ (k) svhn–ResNet, $\hat{\alpha}$ (l) svhn–ResNet, $\langle \log \|\cdot\|_\alpha^\alpha \rangle$

Supplementary Figure 7: Regression plots for model-dataset pairs, based on data from Supplementary Table 3, Supplementary Table 4, and Supplementary Table 5. Each row corresponds to a different dataset-model pair: cifar-100–SENet; cifar-100–WRN; and svhn–ResNet; repsectively. Each column corresponds to a different metric: $\langle \log \|\cdot\|_F \rangle$; $\langle \log \|\cdot\|_\infty \rangle$; $\hat{\alpha}$; and $\langle \log \|\cdot\|_\alpha^\alpha \rangle$; repsectively.

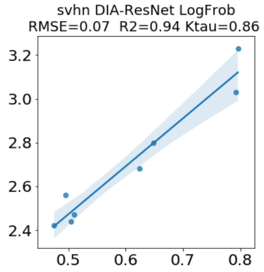

(a) svhn–DIA-ResNet, $\langle \log \|\cdot\|_F \rangle$

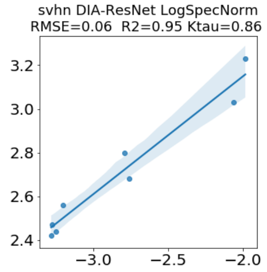

(b) svhn–DIA-ResNet, $\langle \log \|\cdot\|_\infty \rangle$

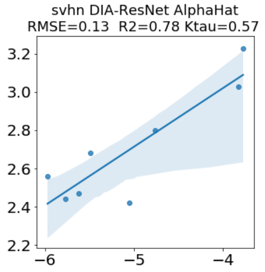

(c) svhn–DIA-ResNet, $\hat{\alpha}$

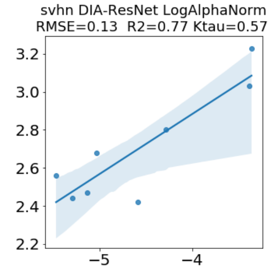

(d) svhn–DIA-ResNet, $\langle \log \|\cdot\|_\alpha^\alpha \rangle$

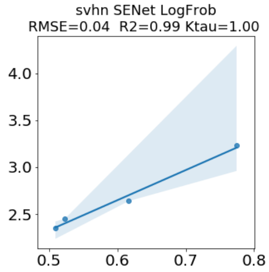

(e) svhn–SENet, $\langle \log \|\cdot\|_F \rangle$

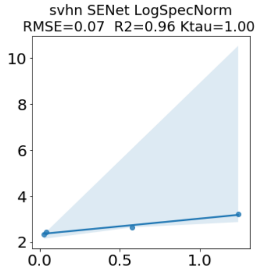

(f) svhn–SENet, $\langle \log \|\cdot\|_\infty \rangle$

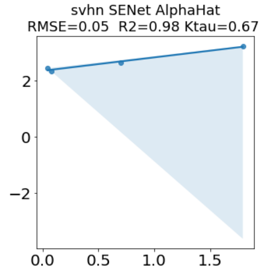

(g) svhn–SENet, $\hat{\alpha}$

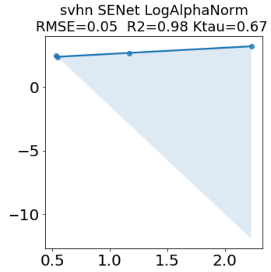

(h) svhn–SENet, $\langle \log \|\cdot\|_\alpha^\alpha \rangle$

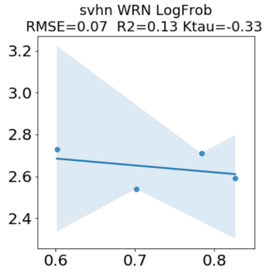

(i) svhn–WRN, $\langle \log \|\cdot\|_F \rangle$

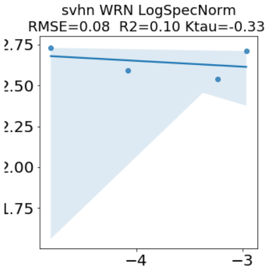

(j) svhn–WRN, $\langle \log \|\cdot\|_\infty \rangle$

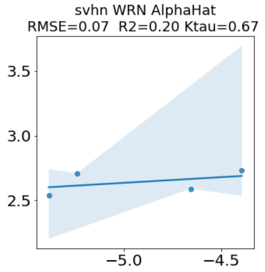

(k) svhn–WRN, $\hat{\alpha}$

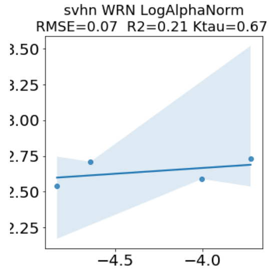

(l) svhn–WRN, $\langle \log \|\cdot\|_\alpha^\alpha \rangle$

Supplementary Figure 8: Regression plots for model-dataset pairs, based on data from Supplementary Table 3, Supplementary Table 4, and Supplementary Table 5. Each row corresponds to a different dataset-model pair: svhn–DIA-ResNet; svhn–SENet; and svhn–WRN; repsectively. Each column corresponds to a different metric: $\langle \log \|\cdot\|_F \rangle$; $\langle \log \|\cdot\|_\infty \rangle$; $\hat{\alpha}$; and $\langle \log \|\cdot\|_\alpha^\alpha \rangle$; repsectively.

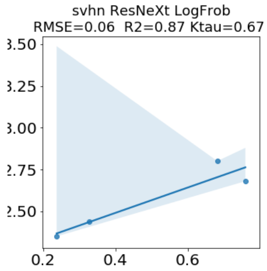

(a) svhn–ResNeXt, $\langle \log \| \cdot \|_F \rangle$

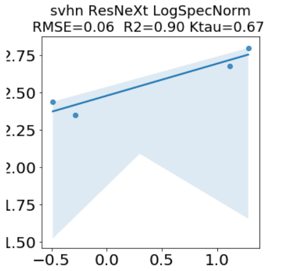

(b) svhn–ResNeXt, $\langle \log \| \cdot \|_\infty \rangle$

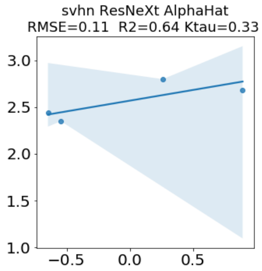

(c) svhn–ResNeXt, $\hat{\alpha}$

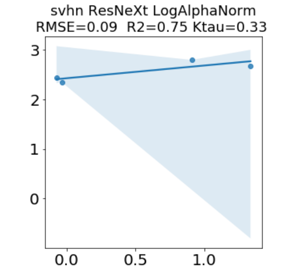

(d) svhn–ResNeXt, $\langle \log \| \cdot \|_\alpha^\alpha \rangle$

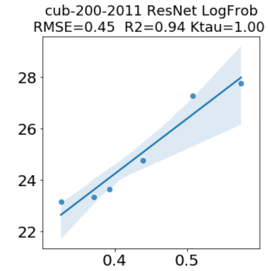

(e) cub-200-2011–ResNet, $\langle \log \| \cdot \|_F \rangle$

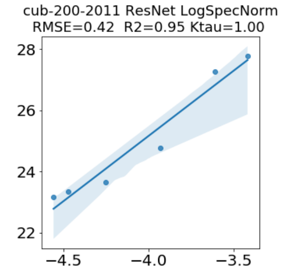

(f) cub-200-2011–ResNet, $\langle \log \| \cdot \|_\infty \rangle$

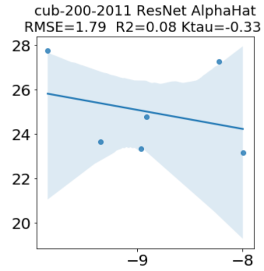

(g) cub-200-2011–ResNet, $\hat{\alpha}$

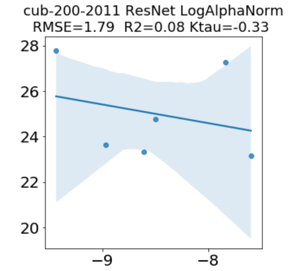

(h) cub-200-2011–ResNet, $\langle \log \| \cdot \|_\alpha^\alpha \rangle$

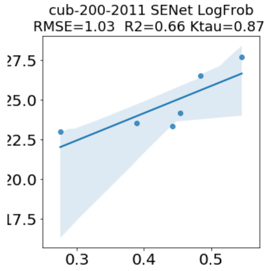

(i) cub-200-2011–SENet, $\langle \log \| \cdot \|_F \rangle$

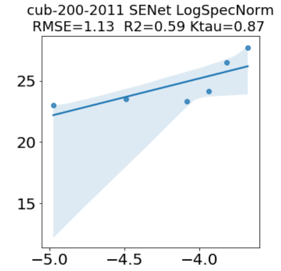

(j) cub-200-2011–SENet, $\langle \log \| \cdot \|_\infty \rangle$

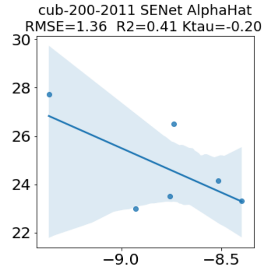

(k) cub-200-2011–SENet, $\hat{\alpha}$

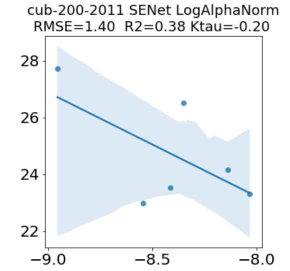

(l) cub-200-2011–SENet, $\langle \log \| \cdot \|_\alpha^\alpha \rangle$

Supplementary Figure 9: Regression plots for model-dataset pairs, based on data from Supplementary Table 3, Supplementary Table 4, and Supplementary Table 5. Each row corresponds to a different dataset-model pair: svhn–ResNeXt; cub-200-2011–ResNet; and cub-200-2011–SENet; repsectively. Each column corresponds to a different metric: $\langle \log \| \cdot \|_F \rangle$; $\langle \log \| \cdot \|_\infty \rangle$; $\hat{\alpha}$; and $\langle \log \| \cdot \|_\alpha^\alpha \rangle$; repsectively.
